# Supplementary material for: Development of an Indirect Enzyme-Linked Immunosorbent Assay Based on the Yeast-Expressed CO-26K-Equivalent Epitope-Containing Antigen for Detection of Serum Antibodies against Porcine Epidemic Diarrhea Virus
Source: Viruses. 2023 Mar 29;15(4):882. doi: 10.3390/v15040882 (PMC10144300; doi:10.3390/v15040882)
Supplement: Supplementary file 1 [file viruses-15-00882-s001.zip › viruses-2148367-supplementary.pdf]

Suppl. Table S1. Information on the S gene of PEDV isolates used in this study

No.	GenBank ID	Virus name	Country-Year	No.	GenBank ID	Virus name	Country-Year
1	KP162057.1	SC1402	China-2014	16	OL762461.1	PEDV TRS	China-2021
2	AF353511.1	Virulent CV777	Belgium-1978	17	OL762459.1	PEDV SD	China-2021
3	KT323979.1	Attenuated CV777	China-1998	18	OL762457.1	PEDV HK	China-2021
4	JQ023162.1	Attenuated DR13	South Korea-2011	19	OL762460.1	PEDV JX	China-2020
5	JN547228.1	CH/S	China-1986	20	OL762458.1	PEDV SDLY	China-2020
6	KP728470.1	SQ2014	China-2014	21	OL762456.1	PEDV SD	China-2020
7	KC189944.1	Attenuated PEDV vaccine	China-2012	22	MT090140.1	SX/YC	China-2019
8	MK138516.1	V7-HB2018	China-2018	23	MT090139.1	HN/XC	China-2019
9	MK140812.1	CH/TP-3-1	China-2018	24	MT090138.1	HN/KF	China-2019
10	MK140811.1	CH/TP-2-2	China-2018	25	MT090137.1	HN/HX	China-2019
11	MK140813.1	CH/TP-4-3	China-2018	26	MN161584.1	SWUN-C6-CH-SCYA	China-2019
12	MH061338.1	CH/SCZY44	China-2017	27	MK820042.1	swun-H3-CH-SCYA	China-2019
13	MH061343.1	CH/SCMY/2018	China-2018	28	MK820041.1	swun-Y1-CH-SCCQ	China-2019
14	MK673545.1	TW/Yunlin550	China-2018	29	MK820040.1	swun-H1-CH-SCYA	China-2019
15	MH061336.1	CH/SCGA	China-2017	30	MK820039.1	swun-MY-CH-SCMY	China-2019

Suppl. Table S1. (continued)

31	MN759311.1	GD-XL	China-2019	43	MH991850.1	S1-GD2018	China-2018
32	MK685665.1	CH-SCNJ	China-2019	44	MH991859.1	X2-GD2018	China-2018
33	MH593896.1	CH/TP/E5	China-2018	45	MN412574.1	SX-WS2/2018	China-2018
34	MH593893.1	CH/TP/4-4	China-2018	46	MN412573.1	SX-WS1/2018	China-2018
35	MK598821.1	CH/SCXC	China-2018	47	MN412572.1	SX-TY2/2017	China-2017
36	MK598819.1	CH/SCZY	China-2018	48	MN412571.1	SX-TY1/2017	China-2017
37	MK592416.1	SWUN2/CH/SCXC	China-2018	49	MN412570.1	HN-XZ/2017	China-2017
38	MK592415.1	SWUN19/CH/SCZY/2018	China-2018	50	MN412567.1	HN-KF/2017	China-2017
39	MN412566.1	HN-YY2/2018	China-2018	51	MH991827.1	B10-GD2017	China-2017
40	MN412565.1	HN-YY1/2018	China-2018	52	MH991842.1	N7-GD2017	China-2017
41	MN412561.1	HN-XC2/2018	China-2018	53	MK532990.1	C1-GD2017	China-2017
42	MH991846.1	PA3-GD2018	China-2018				

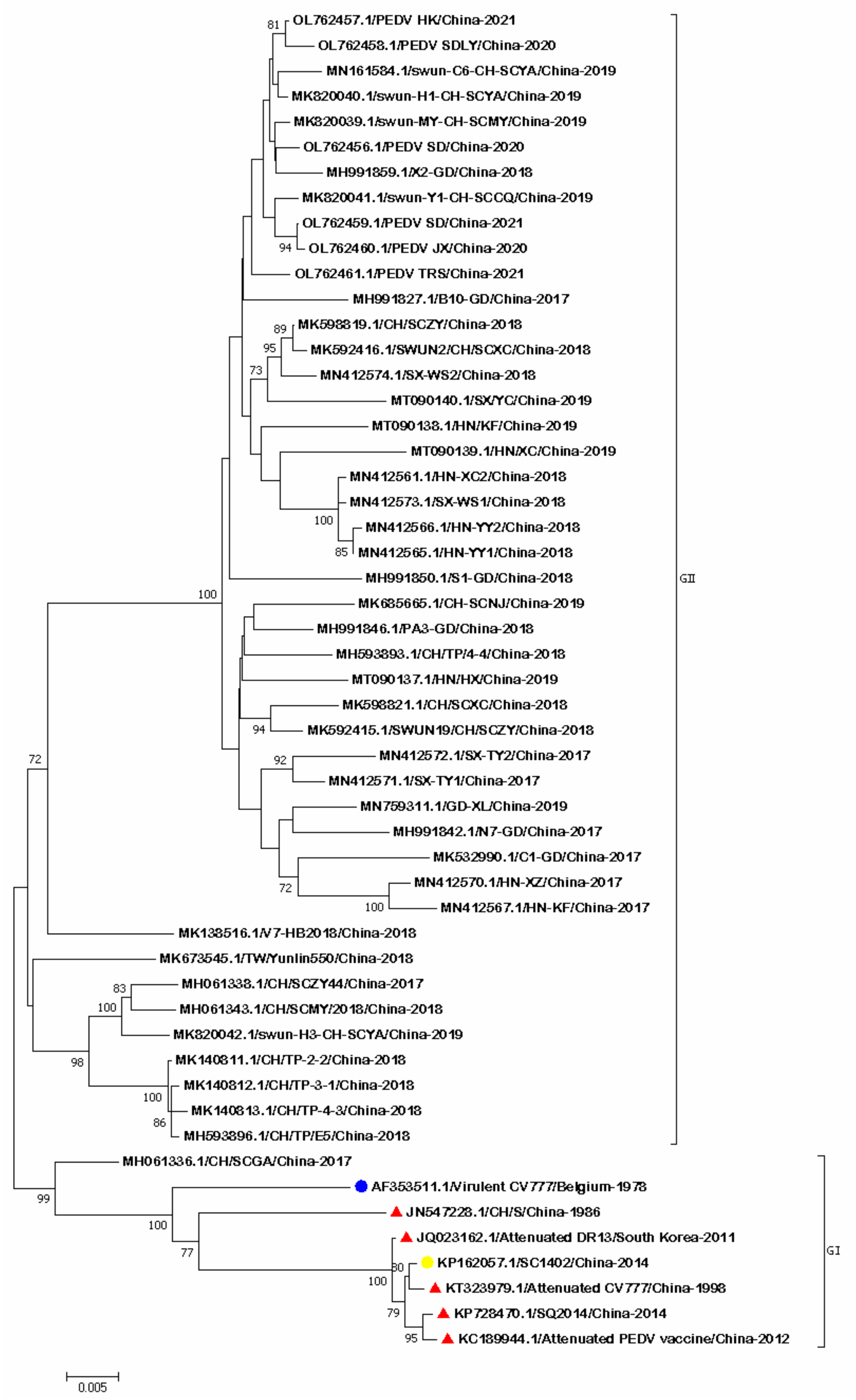

Suppl. Figure S1. Phylogenetic analysis of the full-length S gene of 53 PEDV strains was collected in this study. The numbers at each branch represent bootstrap values greater than 70% of 1000

replicates. The scale bars indicate the number of 0.005 inferred substitutions per site. The blue circle indicates the virulent strain isolated outside of China. The red triangles indicate the cell culture-adapted vaccine strains or the pandemic strains used to make vaccines. The yellow circle indicates the pandemic strain selected in this study.

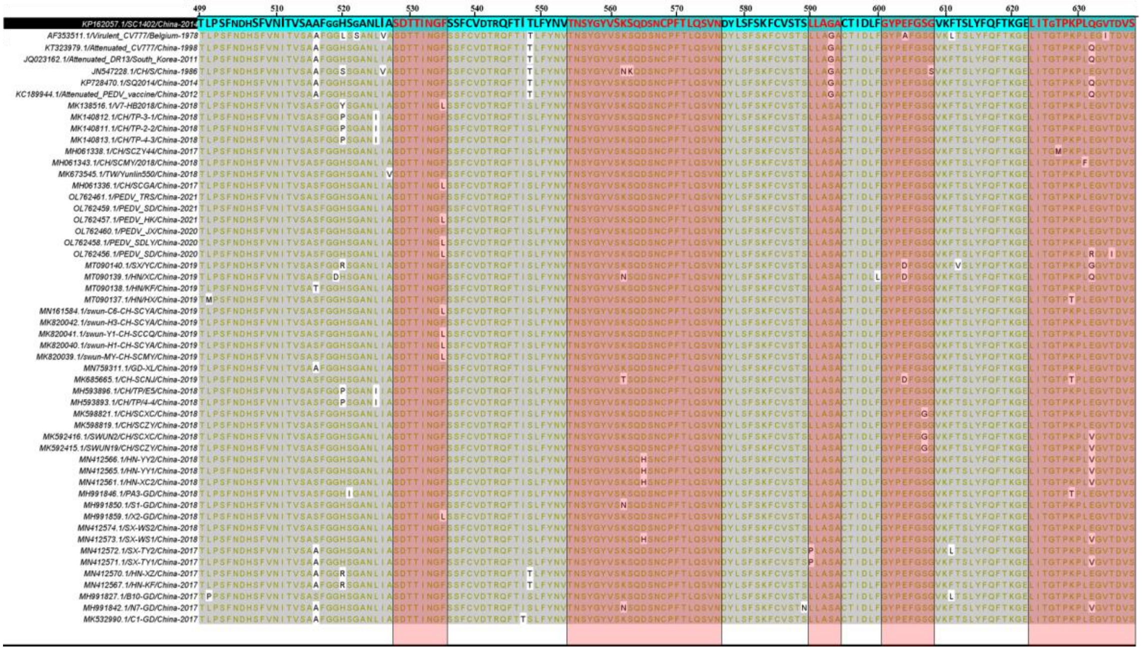

Suppl. Figure S2. Analysis of amino acid mutations in the domain COE of PEDV strains. The amino acid sequences of predicted linear B cell epitopes are shown in ~~red bol~~bold red letters.
